# Expression of an Antiviral Gene *GmRUN1* from Soybean Is Regulated via Intron-Mediated Enhancement (IME)

**DOI:** 10.3390/v13102032

**Published:** 2021-10-08

**Authors:** Pengfei Diao, Hongyu Sun, Zhuo Bao, Wenxia Li, Niu Niu, Weimin Li, Hada Wuriyanghan

**Affiliations:** 1 Key Laboratory of Forage and Endemic Crop Biology, Ministry of Education, School of Life Sciences, Inner Mongolia University, Hohhot 010070, China; lkzxdpf@163.com (P.D.); zixihongyu@163.com (H.S.); nmbzhuo@163.com (Z.B.); liwenxia23@163.com (W.L.); niuniu1901@126.com (N.N.); 2 Biotechnology Research Institute, Chinese Academy of Agricultural Sciences, Beijing 100081, China; liweimin01@caas.cn

**Keywords:** *GmDREB3*, *GmRUN1*, intron-mediated enhancement, resistance gene, salicylic acid, soybean mosaic virus, tobacco mosaic virus, transcriptional regulation

## Abstract

Most of *R* (resistance) genes encode the protein containing NBS-LRR (nucleotide binding site and leucine-rich repeat) domains. Here, *N. benthamiana* plants were used for transient expression assays at 3–4 weeks of age. We identified a TNL (TIR-NBS-LRR) encoding gene *GmRUN1* that was resistant to both soybean mosaic virus (SMV) and tobacco mosaic virus (TMV). Truncation analysis indicated the importance of all three canonical domains for GmRUN1-mediated antiviral activity. Promoter-GUS analysis showed that *GmRUN1* expression is inducible by both salicylic acid (SA) and a transcription factor *GmDREB3* via the cis-elements as-1 and ERE (ethylene response element), which are present in its promoter region. Interestingly, *GmRUN1* gDNA (genomic DNA) shows higher viral resistance than its cDNA (complementary DNA), indicating the existence of intron-mediated enhancement (IME) for *GmRUN1* regulation. We provided evidence that intron2 of *GmRUN1* increased the mRNA level of native gene *GmRUN1*, a soybean antiviral gene *SRC7* and also a reporter gene Luciferase, indicating the general transcriptional enhancement of intron2 in different genes. In summary, we identified an antiviral *TNL* type soybean gene *GmRUN1*, expression of which was regulated at different layers. The investigation of *GmRUN1* gene regulatory network would help to explore the mechanism underlying soybean-SMV interactions.

## 1. Introduction

Two layers of innate immune systems have evolved to recognize the potential pathogens and initiate an effective defense response. The first type of immune response is initiated by the pattern recognition receptors (PRRs) localized at the plasma membrane [1]. PRR recognizes and responds to evolutionarily conserved pathogen-associated molecular pattern (PAMP), and it is called pattern-triggered immunity (PTI) [2]. Some pathogens secrete virulence effectors to counteract PTI. Plants activate the second type of innate immune system to recognize the virulence effectors, named the effector-triggered immunity (ETI) [3]. ETI is generally mediated by a resistance (*R*) gene and leads to local necrosis of plants to limit the continuous spread of pathogens, namely hypersensitive response (HR) [4]. Nucleotide-binding site (NBS), leucine-rich repeat (LRR)-containing proteins (NLRs) occupy the largest proportion in plant R proteins [5]. *NBS-LRR* genes belong to a large gene family, with hundreds of copies in the genome, and are distributed in obvious uneven clusters [6,7]. To date, many *NBS-LRR* type *R* genes have been cloned from different plant species [8].

Plant NLR proteins belong to signal transduction ATPases with numeric domains (STAND) superfamily [9]. The central NBS domain performs the function of molecular switch and controls the binding state of ATP/ADP to mediate downstream signal transduction [10,11]. Leucine-rich repeats (LRRs) are ubiquitously present protein domains involved in mediating protein–protein interactions [12,13]. Some studies suggest that LRR motifs can give recognition specificity in plant defense response [14]. According to their different N-terminal structures, these NBS-LRR proteins can be further divided into two categories: TIR-NBS-LRR (TNL) proteins with the homologous domain of toll/interleukin-1 receptor (TIR) and non-TNL (nTNL) proteins [15]. Most nTNL type-R proteins have a coiled coil (CC) structure at the N-terminal, commonly known as CC-NBS-LRR (CNL) type-R protein [16,17]. Generally, a large number of *CNL* genes are found in all plant genomes, but *TNL* genes are not identified in monocotyledons [18]. The N-terminal CC or TIR domain can be used as a signal transduction center, which is associated with cellular targets or downstream signaling components of effectors [19].

The expression of plant *R* gene needs a strict regulation mechanism, and its ability to activate defense signal and trigger immunity depends on its protein level [20]. Overexpression of *R* gene can lead to autoimmunity and even plant growth retardation [21]. Therefore, the precise regulation of R proteins in plant homeostasis, including transcriptional and translational regulation, is crucial for plant growth and plant disease resistance. The expression of *R* genes is strictly regulated at multiple steps including transcription, post-transcriptional processing, and transcript turnover [22].

Soybean mosaic virus (SMV) is one of the main members of potyvirus [23]. The infection of SMV causes mosaic, necrosis, and other symptoms in many soybean varieties by means of aphid and seed transmission [24]. Therefore, SMV is a major disease that seriously threatens the yield and quality of soybean [25,26,27,28]. SMV genome is a single-stranded sense RNA which encode eleven functional proteins: P1, HC-Pro, P3, PIPO, 6K1, CI, 6k2, NIa-vpg, NIa-Pro, NIb, and CP [29,30]. Three independent SMV resistance loci, Rsv1, Rsv3, and Rsv4, were identified from soybean. Rsv1 is located on chromosome 13, which may contain one or more members of the *NBS-LRR* gene family and is highly resistant to most SMV strains except G7 [31,32]. Rsv3 locus was located on chromosome 14 and was resistant to strains G5, G6, and G7 [33]. Rsv4 is resistant to strains v94-5152 and encodes a SMV specific dsRNase [34,35]. In our previous study, we characterized dozens of SMV-responsive *NLR* genes in the susceptible soybean variety Hefeng25 by transcriptome sequencing [36]. Here, we identified a SMV-resistant soybean gene *GmRUN1* encoding a typical TNL protein. *GmRUN1* also showed resistance to TMV (tobacco mosaic virus) using transient expression assays in *Nicotiana benthamiana*, which is the most widely used experimental host in plant virology, due mainly to the large number of diverse plant viruses that can successfully infect it [37]. *GmRUN1* genomic DNA (gDNA) is more resistant to these two viruses than its cDNA (complementary DNA), indicating the existence of intron-mediated enhancement (IME) for *GmRUN1* regulation. Further analysis showed that intron2 of *GmRUN1* was responsible for transcriptional enhancement of *GmRUN1*. *GmRUN1* expression is also inducible by plant hormone SA and a transcription factor GmDREB3.

## 2. Materials and Methods

### 2.1. Plant Growth Conditions

Soybean [*Glycine max* (L.) Merr.] and *Nicotiana benthamiana* plants were grown in a glasshouse under a 14 h light/10 h dark cycle (24 °C day/22 °C night). Plants were used for transient expression assays at 3–4 weeks of age.

### 2.2. Construction of Recombinant Vectors

To generate cDNA clones of soybean *GmRUN1* and *GmDREB3* genes, total RNA was isolated from soybean cv. Hefeng25 leaves using the TRIzol reagent (Invitrogen, Waltham, MA, USA, Cat#15596026), and cDNA was generated using the GoScript reverse-transcription system (Promega, Madison, WI, USA, Cat#A5001) following the manufacturers’ instructions, and the sequences were then amplified from this cDNA using the primers listed in Supplemental Table S1 with PrimeSTAR^®^ GXL DNA Polymerase (Takara, Kusatsu, Japan, Cat#R050A). For full-length *GmRUN1* gene, PCR product was cloned into the binary vector pBI121 digested by *Sma*I and *Sac*Ι using the In-Fusion HD cloning kit (Takara, Cat#639650). For *GmDREB3* gene, PCR product was cloned into the TA cloning vector using the pMD19-T vector cloning kit (Takara, Cat#3271) and then recombined with the pMD1-T7 vector digested by *BamH*I and *Xho*Ι using the ClonExpress Ultra one-step cloning kit (Vazyme, Nanjing, China, Cat#C115-01). For truncated domain fragments of *GmRUN1* gene, three different domains TIR (1–165 aa), NBS (166–474 aa), LRR (475–1088 aa) were identified by SMART website (http://smart.embl-heidelberg.de/ (Accessed: 1 October 2021)), and the PCR product was cloned into the pCB301-2μ-HDV vector linearized by PCR.

To generate truncated promotor clones of *GmRUN1* gene, genomic DNA was isolated from soybean cv. Hefeng25 leaves using the CTAB method according to the manufacturer’s instructions, and the *GmRUN1* truncated promoter regions were amplified and cloned into the pBI121 vector by replacing the CaMV 35S promoter before the β-glucuronidase (*GUS*) gene.

To generate exogenous intron insert clones of *GmRUN1* gene, the Luciferase gene was cloned from pGWB435-LUC (GenBank No. AB294455.1) and then recombined into the entry vector pHSG299. Four introns of *GmRUN1* gene were amplified from the genomic DNA and cloned into recombined vector pHSG299-LUC digested by *Sca*I site. The recombined entry clones were then recombined with the binary vector pCambia1300 for luciferase assay.

### 2.3. Transient Expression and Virus Inoculation

*Agrobacterium tumefaciens* GV3101 strains carrying recombinant binary vectors wereused to infiltrate *N. benthamiana* leaves. Liquid cultures of all *Agrobacterium* strains were initially grown at 28 °C with agitation in Luria–Bertani (LB) media supplemented with the appropriate antibiotics. The bacterial cells were pelleted by centrifugation at 8000 rpm for 1 min, resuspended in infiltration buffer (10 mM MgCl_2_, 10 mM MES, and 200 μM AS) and adjusted to an appropriate OD600 for infiltration. PJL24 which carried GFP (green fluorescent protein) in TMV genome was used as infectious clones for verification of *GmRUN1* resistance, and GFP fluorescence was detected by a handheld long-wave (365 nm) UV lamp. SMV-N1 strain was used to infect *N. benthamiana* leaves with mechanical inoculation, the SMV-infected leaves under quartz sand grinding in 1×phosphate buffer was daubed to infiltrated site using a writing brush.

### 2.4. Promoter Analysis and In Situ GUS Activity Assay

Promoter elements were predicted for the 3000 bp genomic sequence upstream of *GmRUN1* gene by PlantCare website (http://bioinformatics.psb.ugent.be/webtools/plantcare/html/ (Accessed: 1 October 2021)). The promoter-GUS recombinant vectors, alone or together with the recombinant vectors expressing *GmDREB3* proteins, were infiltrated into *N. benthamiana*. SMV and SA were also applied to the leaves for induction analysis by rub and spray, respectively, and the leaf discs (1 cm in diameter) were cut at 2 dpi (day post inoculation) for GUS staining with X-Gluc as the substrate according to the literature [38].

### 2.5. Luciferase Reporter Assay and Fluorescence Quantitative Analysis

*Agrobacterium* strain GV3101 carrying *LUC* gene inserted different *GmRUN1* introns that recombined in pCambia1300 expression vector were infiltrated into *N. benthamiana* leaves. After 24–48 h, 20 μL 0.5 mM D-luciferin was applied to infiltrated site in the dark. After dark treatment for 3–5 min, the *LUC* expression was detected by CCD imaging system (Berthold Technologies, Bad Wildbad, Germany, LB 985) with IndiGO software at 560 nm for exposure time of 1–3 min, and the fluorescence intensity was visualized to assay the effect of different *GmRUN1* introns. The infiltrated sites of leaves without imaging in the same batch were cut and quick freezing in liquid nitrogen for qRT-PCR analysis. To quantitatively analyze the level of *GmRUN1* resistance, Gel-Pro analyzer software was used for the detection of TMV-GFP intensity in the green channel, and the data were imported to GraphPad Prism7 software to analyze statistically significant and draw graph.

### 2.6. Quantitative Real-Time PCR (qRT-PCR) Analysis

Total RNA extraction and reverse transcription were performed as described above, and the quantitative RT-PCR using gene-specific primers was carried out subsequently in an Analytikjena qPCR instrument using TransStrat^®^ Tip Green qPCR SuperMix (TransGen Biotech, Beijing, China, Cat#AQ141). The data were normalized to *ACTIN* expression by the cycle threshold (CT) 2-ΔΔCT method according to the literature [39] and analyzed by Graphpad Prism7 software. All experiments were repeated at least three times. Primers used in this study are listed in Table S1.

## 3. Results

### 3.1. GmRUN1 cDNA Shows Partial Resistance to TMV and SMV in N. Benthamiana Transient Expression Assay

**Figure 1:**
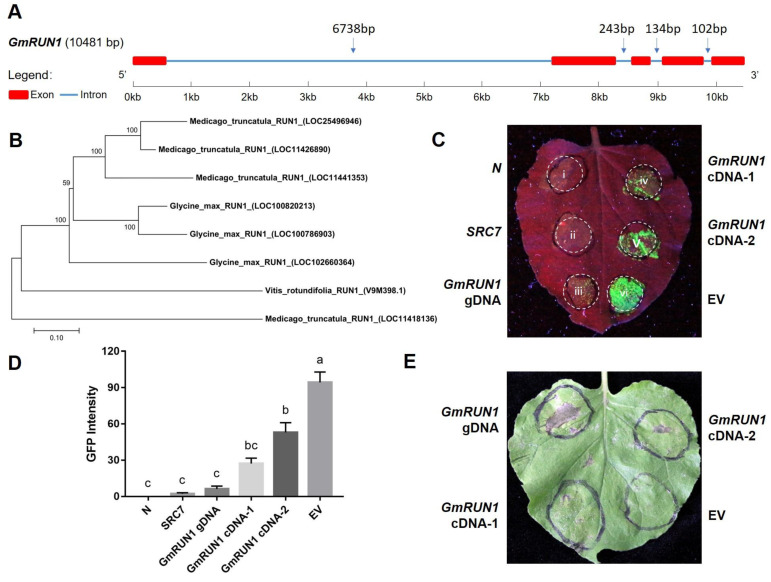
Antiviral activity of *GmRUN1*. (**A**) Gene architecture of *GmRUN1*. Block and line indicate exon and intron, respectively. (**B**) Evolutionary analyses of *GmRUN1*. The evolutionary tree was built using the neighbor-joining method conducted in MEGA7. All positions with less than 50% site coverage were eliminated. (**C**) Transient expression assay for *GmRUN1* antiviral activity for TMV. *N. benthamiana* leaves were infiltrated with *Agrobacterium tumefaciens* GV3101 inocula (OD_600_ = 1.0) carrying different recombinant vectors and co-infected with TMV-GFP. GFP was visualized under hand-held UV lamp (Wavelength = 365 nm) at 5 dpi (days post infiltration). *N*: tobacco N protein. *SRC7*: SMV resistance cluster 7. EV: empty vector. *GmRUN1* cDNA-1: *GmRUN1* cDNA expressed from pCB301 vector. *GmRUN1* cDNA-2: *GmRUN1* cDNA expressed from pBI211 vector. (**D**) Fluorescence quantification of *GmRUN1* transient expression assay. TMV-GFP intensity was analyzed by Gel-Pro analyzer software and normalized against positive control (*N*). (**E**) Transient expression assay for *GmRUN1* antiviral activity for SMV.

Previously, we identified *NBS-LRR* family genes that were involved in SMV–soybean interactions [36]. In the present study, we characterized one such gene with sequence ID of XM_006592417.3 (gene locus: G12g132200) and designated it as *GmRUN1* as it was homologous with *Vitis rotundifolia RUN1* (resistance to *Uncinula Necator* 1) gene (Figure 1A,B) [40,41]. ORF (open-reading frame) sequence or genomic DNA sequence of *GmRUN1* was amplified from soybean cDNA or genomic DNA and were ligated into binary vectors pCB301 and pBI121 to obtain recombinant overexpression vectors. We used *N. benthamiana* transient expression system to investigate the role of *GmRUN1* for SMV resistance. In the parallel experiment, TMV-GFP infectious clone (pJL24) was also used to investigate the antiviral role of *GmRUN1* toward TMV [42]. The above recombinant vectors were transformed into *Agrobacterium* strain GV3101 and transiently expressed in tobacco leaves to detect their resistance to TMV and SMV. In this study, a TMV-resistant tobacco gene *N* [43] and a SMV/TMV resistant soybean gene *SRC7* (unpublished data from the same lab) were used as positive control, while *Agrobacterium* carrying empty vector pBI121 was used as negative control. We defined almost no GFP fluorescence (for TMV) or severe hypersensitive response (HR) (for SMV) as full resistance, weak GFP fluorescence or mild HR as partial resistance, and strong GFP fluorescence or no HR as no resistance. As a result, *GmRUN1* genomic DNA showed full resistance to both TMV and SMV, while its cDNA displayed only partial resistance when it was expressed from either of the binary vectors (Figure 1C–E; Table 1 and Table 2).

### 3.2. Three Canonical Domains of GmRUN1 Are Indispensable for Its Antiviral Activity

**Figure 2:**
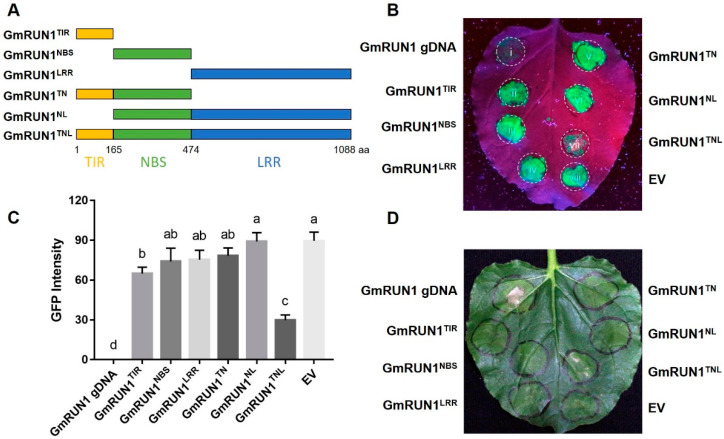
Truncation analysis of *GmRUN1*. (**A**) Domain architecture of *GmRUN1*. Yellow, green, and blue boxes indicate TIR, NBS, and LRR domains, respectively. TN: TIR-NBS, NL: NBS-LRR, TNL: TIR-NBS-LRR. (**B**,**D**) Transient expression assay of different domains for antiviral activity. *N. benthamiana* leaves were infiltrated with *Agrobacterium* GV3101 inocula (OD_600_ = 1.0) carrying different recombinant vectors and co-infected with TMV-GFP (**B**) or SMV (**D**). GFP was visualized under hand-held UV lamp (Wavelength = 365 nm) at 5 dpi (days post infiltration). (**C**) Fluorescence quantification of truncated *GmRUN1* transient expression assay. TMV-GFP intensity was analyzed by Gel-Pro analyzer software and normalized against positive control (*GmRUN1* gDNA).

GmRUN1 is a typical TNL (TIR-NBS-LRR) protein, containing three domains, including a typical N-terminal TIR domain, central NBS domain, and C-terminal LRR domain (Figure 2A). To investigate the importance of these domains, we made truncation analysis. We expressed five truncations for *GmRUN1*, including *GmRUN1*^TIR^, *GmRUN1*^NBS^, *GmRUN1*^LRR^, *GmRUN1*^TN^ (abbreviation for TIR-NBS of *GmRUN1*), and *GmRUN1*^NL^ (abbreviation for NBS-LRR of *GmRUN1*) (Figure 2A). Truncation analysis showed that all of the TIR, NBS, and LRR domains were indispensable for *GmRUN1* antiviral activity, as the deletion of any of them abolished its resistance to both TMV and SMV (Figure 2B–D; Table 3 and Table 4).

### 3.3. GmRUN1 Has IME Phenomena

**Figure 3:**
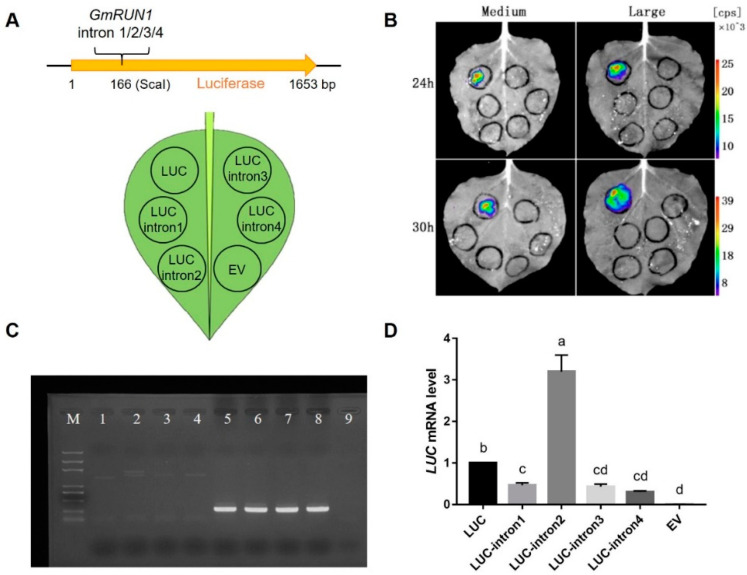
Influence of *GmRUN1* introns insertions on *LUC* reporter gene expression. (**A**) Schematic diagram of *LUC* recombinant vector construction (top panel) and transient expression (bottom panel). (**B**) Luciferase reporter assay of *GmRUN1* introns. *LUC* recombinant vectors with or without different *GmRUN1* introns were transiently expressed in *N. benthamiana* leaves. Images were taken using a Berthold camera 24 and 30 h after infiltration. (**C**) Semiquantitative PCR for transcription assay of *LUC* inserted different *GmRUN1* introns. Lines: (1), *LUC* cDNA, 1653 bp; (2), *LUC*-intron2 cDNA, 1896 bp; (3), *LUC*-intron3 cDNA, 1787 bp; (4), *LUC*-intron4 cDNA, 1755 bp; (5–8), *N. benthamiana ACTIN* of line 1~4; and (9) Negative control. (**D**) qRT-PCR assays of *LUC* mRNA level after inserted with *GmRUN1* introns. Error bars show the SD between biological replicates performed (*n* = 3), and Tukey’s multiple comparisons test was performed between samples in different groups.

**Figure 4:**
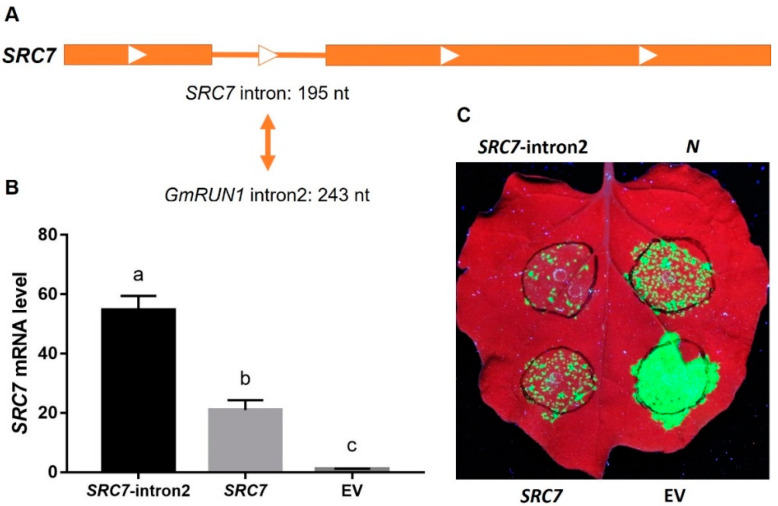
*GmRUN1* intron2 enhances *SRC7* expression. (**A**) Gene architecture of *SRC7*. Block and line indicate exon and intron, respectively. (**B**) qRT-PCR assays of *SRC7* mRNA level after replaced by *GmRUN1* intron2. Error bars show the SD between biological replicates performed (*n* = 3), and Tukey’s multiple comparisons test was performed between samples in different groups. (**C**) Transient expression assay for recombinant *SRC7*-intron2 antiviral activity. *N. benthamiana* leaves were infiltrated with *Agrobacterium tumefaciens* GV3101 inocula carrying different recombinant vectors (OD_600_ = 0.01) and co-infected with TMV-GFP. *N*: tobacco N protein. *SRC7*: SMV resistance cluster 7. *SRC7*-intron2: *SRC7* intron replaced by *GmRUN1* intron2. EV: empty vector.

**Figure 5:**
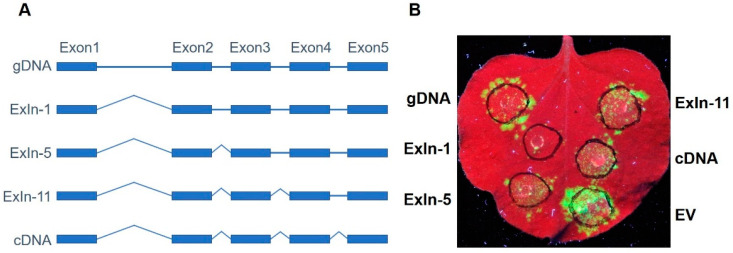
Antiviral activity of *GmRUN1* truncations in different introns. (**A**) Schematic diagram of truncated vector construction with different *GmRUN1* introns. Block and line indicate exon and intron, respectively. (**B**) Transient expression assay of truncated *GmRUN1* introns for antiviral activity. *N. benthamiana* leaves were infiltrated with *Agrobacterium tumefaciens* GV3101 inocula (OD_600_ = 0.5) carrying different recombinant vectors and co-infected with TMV-GFP. GFP was visualized under hand-held UV lamp (Wavelength = 365 nm) at 5 dpi (days post infiltration).

As *GmRUN1* gDNA is resistant to TMV and SMV and its cDNA without the introns has been proved to be partially resistant, we speculate that the introns of *GmRUN1* might be involved in its antiviral activity. *GmRUN1* gDNA contains four introns with the sizes of 6738, 243, 134, and 102 nt, and all of them follow the “GT-AG” rule (Figure 1A). Some introns were reported to enhance gene expression, and this phenomenon was named intron-mediated enhancement (IME) [44,45]. To determine the possible IME of four *GmRUN1* introns, we inserted them at the +166 site of luciferase (*LUC*) reporter gene (Figure 3A). Interestingly, the insertion of any of four introns from *GmRUN1* abolished *LUC* signal (Figure 3B). We speculate that the insertion of these introns may disturb the *LUC* ORF and subsequent protein expression. The PCR amplicons still contained the introns when total RNA was extracted from the inoculated leaves, and *LUC* cDNA was amplified, indicating that the introns were not correctly spliced from *LUC* mRNA (Figure 3C). Therefore, these introns led to insertion mutation in *LUC* gene, showing that the introns of one gene may not be spliced normally when was inserted into other genes. However, we observed that *LUC* mRNA level was significantly upregulated by 3.3 times upon insertion of intron2, showing that intron2 has IME effects at transcriptional level (Figure 3D). We then replaced the intron of soybean antiviral gene *SRC7* with *GmRUN1* intron2 (Figure 4A), and the mRNA level of *SRC7* was increased by 2.6 folds, further demonstrating the IME effect of *GmRUN1* intron2 at the transcriptional level (Figure 4B). Furthermore, the insertion of *GmRUN1* intron2 did not abolish *SRC7* antiviral activity, indicating that *GmRUN1* intron2 was correctly spliced from *SRC7* mRNA (Figure 4C; Table 5). It also indicates that the splicing of intron depends on inserted genes. *SRC7* was homologous to *GmRUN1*; therefore, it is reasonable that *GmRUN1* intron2 can be spliced in *SRC7* but not in nonhomologous *LUC* gene. To further demonstrate the importance of these introns, we made *GmRUN1* expression constructs with truncations in different introns (Figure 5A). The depletion of intron1 increased while further deletion of intron2 decreased antiviral activity, indicating the enhancement of intron2 for antiviral activity (Figure 5B, Table 6). Taken together, *GmRUN1* intron2 has an IME effect.

### 3.4. GmRUN1 Expression Is Transcriptionally Regulated by SA

**Figure 6:**
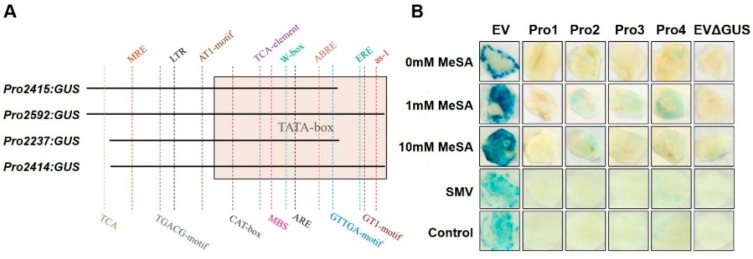
Transcriptional regulation of *GmRUN1* promoter. (**A**) Schematic diagram of truncated vector construction with different region of *GmRUN1* promoter. Colored dashed lines and boxes indicate cis-acting regulatory element predicted by PlantCare database. (**B**) *GUS* activity assay of different region of *GmRUN1* promoter. *GUS* activity was detected at 3 days post SA or SMV induction. EV: pBI121 empty vector. Pro1~4: *Pro2415:GUS, Pro2592:GUS, Pro2237:GUS,* and *Pro2414:GUS*. EVΔGUS: pBI121 empty vector removed *GUS* gene.

Having confirmed the IME effect of intron2 on *GmRUN1* expression, we next intended to clone the *GmRUN1* promoter and further examine the transcriptional regulation of *GmRUN1*. We used GUS reporter to assess promoter activity. The promoter region of *GmRUN1* was amplified from soybean genomic DNA and was cloned into binary vector pBI121 to obtain *Pro:GUS* reporter vectors. We made four different *Pro:GUS* constructs, harboring different lengths of the promoter region, namely *Pro2415:GUS*, *Pro2592:GUS*, *Pro2237:GUS*, and *Pro2414: GUS* (Figure 6A). When these *Pro:GUS* constructs are transiently expressed in *N. benthamiana*, they did not show any GUS signal, demonstrating that they possessed very low basal transcriptional activity (Figure 6B). The infection of SMV did not induce the GUS expression in any of the *Pro:GUS* constructs, indicating that *GmRUN1* expression is not inducible by SMV infection (Figure 6B). Salicylic acid (SA) is a well-known defense hormone which is generally implicated in plant immunity against plant viruses including SMV [36]. Treatment with MeSA, an analog of SA, elevated GUS expression for the *Pro2592:GUS* and *Pro2414:GUS* reporters but not in *Pro2415:GUS* and *Pro2237:GUS* reporters (Figure 6B).

### 3.5. GmRUN1 Expression Is Transcriptionally Induced by Transcription Factor GmDREB3

**Figure 7:**
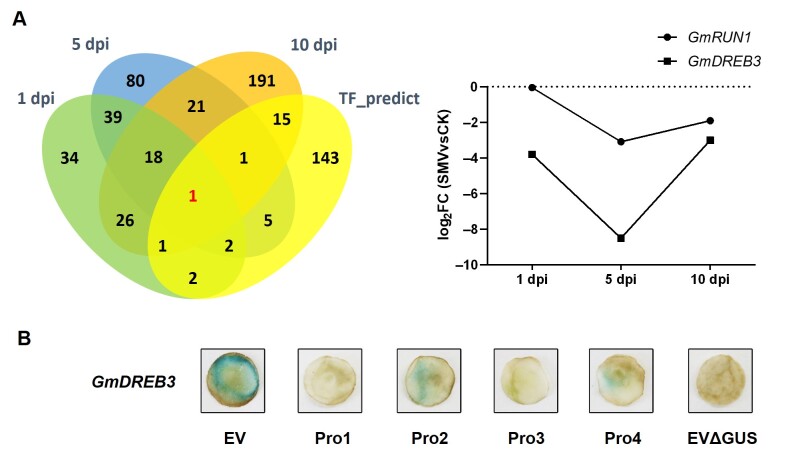
*GmDREB3* positively regulates *GmRUN1*. (**A**) Venn diagrams of predicted TFs transcription level consistent with *GmRUN1* at 1/5/10 dpi (left panel). Red number indicates *GmDREB3* which shows consistent expression pattern with *GmRUN1* in graph at 1/5/10 dpi (right panel). (**B**) *GUS* activity assay of different region of *GmRUN1* promoter coexpressed with *GmDREB3*. *GUS* activity was detected at 3 dpi. EV: pBI121 empty vector. Pro1~4: *Pro2415:GUS, Pro2592:GUS, Pro2237:GUS,* and *Pro2414:GUS*. EVΔGUS: pBI121 empty vector removed *GUS* gene.

Based on the above results, we further analyzed cis-regulatory element present in *GmRUN1* promoter using PlantCare website (http://bioinformatics.psb.ugent.be/webtools/plantcare/html/ (Accessed: 1 October 2021)). Interestingly, we found two cis-elements immediately upstream of *GmRUN1* ORF which were present in SA-inducible constructs *Pro2592:GUS* and *Pro2414:GUS,* while lacking in SA noninducible *Pro2415:GUS* and *Pro2237:GUS* constructs (Figure 6A). One *cis* element is the SA-responsive as-1, which might contribute to SA induction of *GmRUN1* promoter. Another *cis* element is the ERF binding element ERE, which is close to as-1 element on *GmRUN1* promoter (Table 7). Previously, we showed that *GmRUN1* expression is repressed upon SMV infection in soybean [36]. From our RNA-seq data, we also found that *GmDREB3* is significantly repressed by SMV infection, and *GmDREB3* expression pattern is positively correlated with that of *GmRUN1* (Figure 7A). The data lead us to the assumption that *GmDREB3* might be a positive regulator of *GmRUN1*. Coexpression of *GmDREB3* elevated GUS expression for the *Pro2592:GUS* and *Pro2414:GUS* reporters but not in *Pro2415:GUS* and *Pro2237:GUS* reporters, further demonstrating positive regulation of *GmRUN1* expression by *GmDREB3* via the ERE cis element at *GmRUN1* promoter (Figure 7B).

## 4. Discussion

In this study, we identified an antiviral gene *GmRUN1* from soybean. *GmRUN1* encodes a typical TIR-NBS-LRR protein and gives resistance to both a *Potyvirus* SMV and a *Tobamovirus* TMV. Truncation analysis showed that the all of the canonical domains TIR, NBS, and LRR were necessary for GmRUN1 antiviral activity. In some reports, TIR-NBS domains but not LRR domain were sufficient to trigger immune responses, especially in transient expression assay. For example, *Arabidopsis* powdery mildew resistance gene *TN2* and autoimmune-related gene *CHS1* encode functional TIR-NBS proteins [46,47]. Furthermore, *TIR-NBS* genes were broadly reported in variety of plant species, including the leguminous plants such as soybean and common bean [48,49]. Therefore, the antiviral mechanism of *GmRUN1* might be different from those of *TIR-NBS* genes and deserves further investigation.

Overexpression of *GmRUN1* showed HR upon infection with SMV. Cell-death-triggering activity of R proteins should be under strict control so as to trigger timely immune response only upon pathogen infection and to also avoid fitness costs at pathogen-free conditions [50]. Therefore, multiple layers of regulation at transcriptional, post-transcriptional, and protein activity levels exist for expression control of *R* genes [22]. The expression of *GmRUN1* is inducible by the treatment with major defense hormone SA. Promoter truncation analysis narrowed down the SA-responsive element to a −326~−336 nt (10 nt) region, where we identified a SA-responsive *cis* element as-1, which is most likely contributed to SA induction on *GmRUN1* expression [51,52]. SA is an important defense hormone which contributes to the immunity against various pathogens, especially plant viruses, and leads to systemic acquired resistance (SAR) [53]. For example, SA was reported to be involved in defense response mediated by *R* genes such as tobacco *N* and *Arabidopsis AtTN10* [48,54]. Besides this as-1 element, we also found a ERE element in the SA-responsive promoter region. A putative transcription factor GmDREB3 upregulates *GmRUN1* promoter activity via ERE-dependent manner, and the expression of *GmRUN1* is positively correlated with *GmDREB3* expression, demonstrating that *GmRUN1* expression is transcriptionally induced by GmDREB3. The effects of *DREB* on *R* gene expression and involvement in plant immunity were also observed in other plant species [55,56,57].

It has been shown that some introns had positive regulatory roles on gene expression, but some others possessed inhibitory effects [58]. In 1987, Callis et al. first discovered that introns can mediate the enhancement of gene expression in maize cells [44]. Subsequently, the phenomenon of IME was observed in mammals, nematodes, and yeasts [59,60,61]. Some endogenous introns can compensate for the low-level expression driven by the weak promoter [62]. IME is also associated with specific sequence motifs, such as TTNGATYTG and CGATT [63]. Several introns were also shown to have both promoter activity and enhancer function [64]. In most studies, IME was attributable to increased mRNA accumulation at the transcriptional level; however, there are also some data that provide evidence of enhanced translation [65,66]. Here, we provided evidence that intron2 of *GmRUN1* increased mRNA level of native gene *GmRUN1*, a homologous gene *SRC7* and also a reporter gene *Luciferase*, indicating the general transcriptional enhancement of this intron2 in different genes. However, we also showed that correct splicing of intron2 might depend on the inserted genes. The in-depth study of IME phenomenon provides a basis for the wide application of functional introns.

## 5. Conclusions

Plant viruses pose threats to agriculturally important crops. SMV is a major pathogen of soybean and causes heavy yield losses worldwide. Although several *R* genes have been cloned from multiple host varieties, soybean–SMV interactions are still elusive. In this study, we identified a TNL (TIR-NBS-LRR)-type antiviral gene *GmRUN1* from soybean. *GmRUN1* genomic DNA showed full resistance to both TMV and SMV, while its cDNA displayed only partial resistance, and three canonical domains of *GmRUN1* are indispensable for its antiviral activity. *GmRUN1* is spectacular as its expression is regulated at multiple layers, such as SA induction, GmDREB3 transcriptional activation, and IME from intron2. *GmRUN1* represents a novel SMV-resistance gene and deserves further functional study.

## Figures and Tables

**Table 1:** Phenotypic statistics of TMV appearance upon transient expression of different genes.

Gene Name	Full Resistance	No Resistance	Partial Resistance
pBI121-*N*	30/30 (100%)	0/30 (0%)	0/30 (0%)
pBI121-*SRC7*	30/30 (100%)	0/30 (0%)	0/30 (0%)
pBI121-*GmRUN1* gDNA	24/30 (80%)	0/30 (0%)	6/30 (20%)
pCB301-*GmRUN1* cDNA	0/30 (0%)	13/30 (43%)	17/30 (57%)
pBI121-*GmRUN1* cDNA	0/30 (0%)	24/30 (80%)	6/30 (20%)
pBI121	0/30 (0%)	30/30 (100%)	0/30 (0%)

**Table 2:** Phenotypic statistics of SMV upon transient expression of different genes.

Gene Name	HR/Total Leaves
pBI121-*GmRUN1* gDNA	14/30 (47%)
pCB301-*GmRUN1* cDNA	9/30 (30%)
pBI121-*GmRUN1* cDNA	7/30 (23%)
pCB301	0/30 (0%)

**Table 3:** Phenotypic statistics of TMV appearance upon transient expression of different genes.

Gene Name	Full Resistance	No Resistance	Partial Resistance
pBI121-*GmRUN1* gDNA	17/25 (68%)	0/25 (0%)	8/25 (32%)
pCB301-*GmRUN1^TIR^*	0/25 (0%)	25/25 (100%)	0/25 (0%)
pCB301-*GmRUN1^NBS^*	0/25 (0%)	25/25 (100%)	0/25 (0%)
pCB301- *GmRUN1^LRR^*	0/25 (0%)	25/25 (100%)	0/25 (0%)
pCB301-*GmRUN1^TN^*	0/25 (0%)	25/25 (100%)	0/25 (0%)
pCB301-*GmRUN1^NL^*	0/25 (0%)	25/25 (100%)	0/25 (0%)
pCB301-*GmRUN1^TNL^*	0/25 (0%)	6/25 (24%)	19/25 (76%)
pCB301	0/25 (0%)	25/25 (100%)	0/25 (0%)

**Table 4:** Phenotypic statistics of SMV upon transient expression of different genes.

Gene Name	HR/Total Leaves
pBI121-*GmRUN1* gDNA	10/25 (40%)
pCB301-*GmRUN1^TIR^*	1/25 (4%)
pCB301-*GmRUN1^NBS^*	0/25 (0%)
pCB301-*GmRUN1^LRR^*	0/25 (0%)
pCB301-*GmRUN1^TN^*	1/25 (4%)
pCB301-*GmRUN1^NL^*	0/25 (0%)
pCB301-*GmRUN1^TNL^*	6/25 (24%)
pCB301	0/25 (0%)

**Table 5:** Phenotypic statistics of TMV appearance upon transient expression of different genes.

Gene Name	Full Resistance	No Resistance	Partial Resistance
pCambia1300-*SRC7*-intron2	15/20 (75%)	0/20 (0%)	5/20 (25%)
pCambia1300-*SRC7*	7/20 (35%)	0/20 (0%)	13/20 (65%)
pCambia1300-*N*	8/20 (40%)	0/20 (0%)	12/20 (60%)
pCambia1300	0/20 (0%)	20/20 (100%)	0/20 (0%)

**Table 6:** Phenotypic statistics of TMV appearance upon transient expression of different genes.

Gene Name	Full Resistance	No Resistance	Partial Resistance
pCB301-gDNA	9/35 (26%)	0/35 (0%)	26/35 (74%)
pCB301-*GmRUN1*-ExIn-1	15/35 (43%)	0/35 (0%)	20/35 (57%)
pCB301-*GmRUN1*-ExIn-5	2/35 (6%)	0/35 (0%)	33/35 (94%)
pCB301-*GmRUN1*-ExIn-11	2/35 (6%)	5/35 (14%)	28/35 (80%)
pCB301-cDNA	0/35 (0%)	17/35 (49%)	18/35 (51%)
pCB301	0/35 (0%)	35/35 (100%)	0/35 (0%)

**Table 7:** Promoter elements and their functions of *GmRUN1*.

Promoter Element	Sequence	Function
TATA-box	TATAA	Core promoter element around –30 of transcription start
CAAT-box	CCAAT	Common cis-acting element in promoter and enhancer regions
CGTCA-motif	CGTCA	Cis-acting regulatory element involved in the MeJA-responsiveness
TGACG-motif	TGACG	Cis-acting regulatory element involved in the MeJA-responsiveness
as-1	TGACG	Cis-acting element related to salicylic acid induction
TCA-element	TCAGAAGAGG	Cis-acting element involved in salicylic acid responsiveness
TCA	TCATCTTCAT	Unknown functional element
ABRE	ACGTG	Cis-acting element involved in the abscisic acid responsiveness
ERE	ATTTTAAA	Cis-acting element involved in ethylene response
ARE	AAACCA	Cis-acting regulatory element essential for the anaerobic induction
LTR	CCGAAA	Cis-acting element involved in low-temperature responsiveness
MBS	CAACTG	MYB binding site involved in drought inducibility
W-box	TTGACC	Cis-acting element involved in disease resistance
CAT-box	GCCACT	Cis-acting regulatory element related to meristem expression
AT1-motif	AATTATTTTTTATT	Part of a light-responsive module
GT1-motif	GGTTAAT	Light-responsive element
TCT-motif	TCTTAC	Part of a light-responsive element
G-Box	CACGTG	Cis-acting regulatory element involved in light responsiveness
MRE	AACCTAA	MYB binding site involved in light responsiveness
Box 4	ATTAAT	Part of a conserved DNA module involved in light responsiveness

## Data Availability

Not applicable.

## Supplementary Materials

The following are available online at www.mdpi.com/article/10.3390/v13102032/s1, Table S1: Primers used in this study.
