# Supplementary material for: Expression of an Antiviral Gene GmRUN1 from Soybean Is Regulated via Intron-Mediated Enhancement (IME)
Source: Viruses. 2021 Oct 8;13(10):2032. doi: 10.3390/v13102032 (PMC8539222; doi:10.3390/v13102032)
Supplement: Supplementary file 1 [file viruses-13-02032-s001.zip › viruses-1382287-supplementary.pdf]

Supplementary data legends.

Supplementary Table S1. Primers used in this study.

Primers	Sequence	Description
RUN-F	ctctagaggatccccgggTAGCTTTCGCTGTTGCAATTG	*GmRUN1* clone
RUN-R	gatcggggaaattcgagctcGGACAAATATGGAAGCCATTA	
Ex-1F	gttcatttcatttggagaggATGGCTTCCAACAGCATGGT	
Ex-1R	CAATCTCATCATATTGTGACTTATTCATCATATCCCAACC	
Ex-2F	GTCACAATATGATGAGATTGAAAAA	
Ex-2R	GCTTCATTGTTTGTAGTTTCCGTTGTCTTCGACATATCAT	
Ex-3F	GAAACTACAAACAATGAAGCCATTG	*GmRUN1* exon clone
Ex-3R	GCTCTTAAATTTGGCAGATACTTTATGCCTTTCCATAGTT	
Ex-4F	TATCTGCCAAATTTAAGAGCTTTGG	
Ex-4R	GGGTAGCTGACTCTTGGCTCACCTGCAGGATTTGTAACAA	
Ex-5F	GAGCCAAGAGTCAGCTACCCCTATT	
Ex-5R	tggagatgccatgccgacccCTAGCTTCCAGCTTCTAATG	
Pro-1F	gaccatgattacgccaagcttTCTCTTCATGGCTTATTGAACA	*GmRUN1* promoter clone
Pro-1R	ggactgaccacccggggatccTTCGAACCATCGTGTACCAA	
Pro-2F	gaccatgattacgccaagcttTGAAAGACAATGAGAAGGATGA	
Pro-2R	ggactgaccacccggggatccCGAAATTGTGGGTTGTGATG	
TIR-F	gttcatttcatttggagaggATGGCTTCCAACAGCATG	
TIR-R	tggagatgccatgccgacccTTAagcgtaatctggtacgtcgtatgggtaATCCCAACCAGAGAA	
NBS-F	gttcatttcatttggagaggATGATGAATAAGTCACAA	*GmRUN1* domain clone
NBS-R	tggagatgccatgccgacccTTAagcgtaatctggtacgtcgtatgggtaAATTTCAGCATGAAA	
LRR-F	gttcatttcatttggagaggATGGGAATAAGAGTTCTC	
LRR-R	tggagatgccatgccgacccCTAagcgtaatctggtacgtcgtatgggtaGCTTCCAGCTTCTAA	
LUC-1F	aaaacgacggccagtgaattcATGGAAGACGCCAAAAACATAAA	
LUC-1R	gccaagcttgcatgcctgcagTTACACGGCGATCTTTCCGC	*LUC* clone
LUC-2F	agagaacacgggggacgagctcATGGAAGACGCCAAAAACATAAAG	
LUC-2R	cgatttgttggatcccgggtaccTTACACGGCGATCTTTCCG	
In-1F	acatcacttacgctgagtactGTAAGTAATATTCTGTAATACTAATGAAAAGTCATG	
In-1R	gaacggacatttcgaagtactCTGAAAGTTTAAAGAATAGCAAAAGTACC	
In-2F	acatcacttacgctgagtactGTATGTATTGTTACAAAATACAAGATTTTATTAAT	
In-2R	gaacggacatttcgaagtactCTAAAGCATTACAAAATGATTCATAAATTG	*LUC*-intron clone
In-3F	acatcacttacgctgagtactGTATCTTTTTAATTTTACTTTTTGAAATGAGT	
In-3R	gaacggacatttcgaagtactCTGTAAAATACCAACAAGAATGAAGAGA	
In-4F	acatcacttacgctgagtactGTACTCACTCTATTCGATCTTCCCTACA	
In-4R	gaacggacatttcgaagtactCTGGTACATGAAACATGGACAGGT	
SRC7-1F	gagaacacgggggacgagctcATGGCTGCAACAACACGTTCC	
SRC7-1R	CCATCTTTGAAATGATGGCCAGACAAG	
Ni2-F	tggccatcatttcaaagatggGTATGTATTGTTACAAAATACAAG	*SRC7*-intron2 clone
Ni2-R	aacttgtattcatatgcatctCTAAAGCATTACAAAATGATTC	
SRC7-2F	AGATGCATATGAATACAAGTTTATTGG	
SRC7-2R	atttgttggatcccgggtaccTTAGGCTAGATTGCCATACTGG	
Actin-qL	GTCAAGGCTGGGTTTGCTGG	qPCR for *ACTIN*
Actin-qR	CCCACGTAGGCATCTTTCTG	
LUC-qF	GACCAACGCCTTGATTGACA	qPCR for *LUC*
LUC-qR	AGCCACCTGATAGCCTTTGT	
SRC7-qF	AAGCTTCACGGTGGAGATGA	qPCR for *SRC7*
SRC7-qR	ACCAACAGCCCTTCACTCTT	
DREB-F	gactctagaggatccATGGCGAAACCCAGCAG	*GmDREB3* clone
DREB-R	tgaagcctcctcgagTCAAAAATTCCACAAGAAAGATTC	
